# Priming and the guidance by visual and categorical templates in visual search

**DOI:** 10.3389/fpsyg.2014.00148

**Published:** 2014-02-24

**Authors:** Anna Wilschut, Jan Theeuwes, Christian N. L. Olivers

**Affiliations:** Department of Cognitive Psychology, VU UniversityAmsterdam, Netherlands

**Keywords:** visual search, priming, working memory, template, cueing, SOA

## Abstract

Visual search is thought to be guided by top-down templates that are held in visual working memory. Previous studies have shown that a search-guiding template can be rapidly and strongly implemented from a visual cue, whereas templates are less effective when based on categorical cues. Direct visual priming from cue to target may underlie this difference. In two experiments we first asked observers to remember two possible target colors. A postcue then indicated which of the two would be the relevant color. The task was to locate a briefly presented and masked target of the cued color among irrelevant distractor items. Experiment 1 showed that overall search accuracy improved more rapidly on the basis of a direct visual postcue that carried the target color, compared to a neutral postcue that pointed to the memorized color. However, selectivity toward the target feature, i.e., the extent to which observers searched selectively among items of the cued vs. uncued color, was found to be relatively unaffected by the presence of the visual signal. In Experiment 2 we compared search that was based on either visual or categorical information, but now controlled for direct visual priming. This resulted in no differences in overall performance nor selectivity. Altogether the results suggest that perceptual processing of visual search targets is facilitated by priming from visual cues, whereas attentional selectivity is enhanced by a working memory template that can formed from both visual and categorical input. Furthermore, if the priming is controlled for, categorical- and visual-based templates similarly enhance search guidance.

## Introduction

Most visual behavior occurs in the context of a multitude of stimuli at different spatial locations. Often we need to search through these stimuli in order to first find, and then focus on the information that is relevant. Such visual search is thought to be guided by the top-down goals of the observer, which shape the *templates* that are held in visual working memory (WM; Duncan and Humphreys, 1989; Bundesen, 1990; Wolfe, 1994; Desimone and Duncan, 1995). Thus, templates are the representations defining what the observer is looking for. For understanding visual search it is important to investigate the temporal dynamics of the template. How fast is it set up and implemented? These dynamics are likely to depend on the type of information that is used for forming the template.

The time course of visual search guidance has been studied previously by cued visual search tasks (Meyers and Rhoades, 1978; Wolfe et al., 2004; Vickery et al., 2005; Schmidt and Zelinsky, 2011; Knapp and Abrams, 2012; Wilschut et al., 2013). In these tasks a centrally presented cue indicates the target identity at variable intervals before the appearance of the search display. The task is to find the target and then respond on the basis of its presence or properties. The efficacy of visual search guidance can then be measured as a function of the time between the cue and target display (SOA). As a general rule, performance improves up to an optimal level within a few 100 ms from the cue, which is then assumed to reflect the time that it takes to implement a template from WM.

One focus in the cued search studies has been how visual search can be guided as based on cues that are presented in different modalities (Meyers and Rhoades, 1978; Wolfe et al., 2004; Vickery et al., 2005; Schmidt and Zelinsky, 2011; Knapp and Abrams, 2012). The underlying idea has been that a picture of the target leads directly to the creation of a visual WM template that can then effectively guide visual search. In contrast, cueing the search by one or more words describing the target may lead to different types of templates. Such categorical cues could be first transformed to a visual template, or alternatively, search may in itself be guided by categorical information (Lupyan and Spivey, 2010; Lupyan and Thompson-Schill, 2012). So far, studies that have manipulated the cue modality and the SOA have found that visual cues lead to a rapid performance enhancement within typically around 400 ms (but up to 800 ms depending on the specific study). By contrast, categorical cues were always found to be less efficient, resulting in overall lower performance. In terms of the time course the effects of categorical cues were more mixed, and some studies seem to suggest a relatively similar timing for visual and categorical cues (Meyers and Rhoades, 1978; Wolfe et al., 2004), whereas others find that the maximal enhancement by categorical cues occurs considerably later in time compared to visual cues (Vickery et al., 2005; Schmidt and Zelinsky, 2011; Knapp and Abrams, 2012). Based on these results it has been suggested that visual search can be guided efficiently by templates that are based on visual information, but that guidance is slow and inefficient when templates are based on categorical information, because it takes time to encode this into a visual template, or because a categorical template is in itself less effective.

Even though a number of studies have supported the idea that visual search is guided rapidly and effectively only by visual-based templates in WM (Meyers and Rhoades, 1978; Wolfe et al., 2004; Vickery et al., 2005; Schmidt and Zelinsky, 2011; Knapp and Abrams, 2012; see also Theeuwes and Van der Burg, 2011), there could be an alternative explanation for these findings. Importantly, the effective cue types in these studies were always identical pictures of the target. While it has been assumed that pictorial cues are encoded into templates in visual WM, which then guide visual search, it is also possible that such cues activate another memory system, namely *implicit priming* (Maljkovic and Nakayama, 1994; Kristjánsson and Campana, 2010). It is important to differentiate between the two memory types, not only because virtually all theories of visual attention assume a strong role for WM in guiding visual search (Duncan and Humphreys, 1989; Bundesen, 1990; Wolfe, 1994; Desimone and Duncan, 1995), but also because they differ in terms of how they operate. While visual WM representations are thought to be controlled by the intentions of the observer, the representations in implicit memory are typically considered to be formed in a more automatic fashion (Wolfe et al., 2003; Theeuwes et al., 2006; Leonard and Egeth, 2008). Some evidence for inter-trial priming was found in the study of Wolfe et al. (2004), as the RTs were faster in trials in which the target was repeated. Interestingly, the inter-trial priming effect was more pronounced in the word cue condition relative to the picture cue condition, presumably because in the latter the cue acted as a prime itself, and thus the effect of repetition between trials was drowned. This further increases the possibility that the advantage by visual cues may be at least partially due to an automatic type of facilitation rather than due to WM (see also Vickery et al., 2005; for a proposal that cue-target priming may play a role).

**Figure 1:**
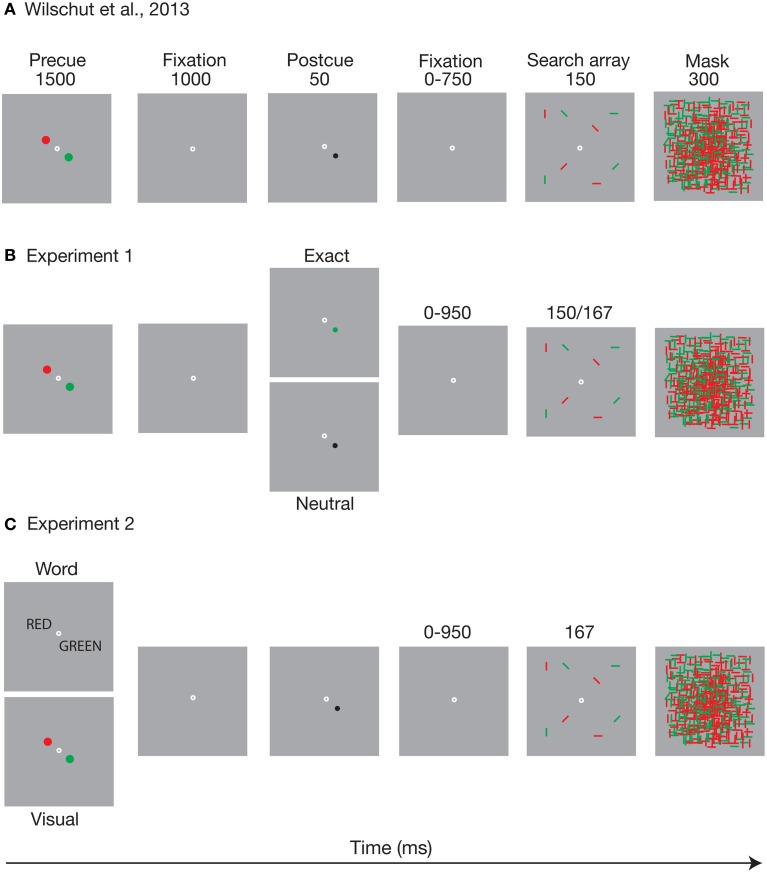
**The figure depicts an outline of the task of (A) Wilschut et al. (2013) as well as (B) the postcue manipulation, and (C) the precue manipulation of Experiments 1, 2 of the present study, respectively. (A)** adapted from Wilschut et al. (2013). The time it takes to turn a memory into a template. Copyright by the Association for Research in Vision and Ophthalmology (ARVO).

In our recent study (Wilschut et al., 2013) we used a procedure that deviated somewhat from the standard way of cueing visual search. We did this to (a) investigate the time course of templates based on a memory of a visual stimulus feature, rather than on a picture of that stimulus, and to (b) control for direct priming from such a visual stimulus feature. Instead of varying the time between the specific target image cue and the search array, we first required observers to load two potential target colors (red and green) into memory. We then presented a visually neutral postcue that indicated which specific color had to be selected from memory, but that did not contain that color itself (i.e., the cue was black). Figure 1A presents an outline of the task. Observers first memorized the two possible target colors, after which a spatial postcue indicated which of these was relevant for the trial, and thus which memory should be turned into a template. Because the postcue was neutral in terms of the target color, direct priming was controlled for. The time between the postcue and the search array was then varied, to measure the time course of search guidance on the basis of the activated WM template.

Moreover, the set sizes of the relevant and irrelevant color in the search display were varied independently, so that the influence of items in the cued vs. uncued color could be used as a direct index of selectivity (cf. Egeth et al., 1984; Kaptein et al., 1995; Donk and Theeuwes, 2001). The more selective people are with regards to the target color, the more search should be affected by the items sharing that color, and the less they should be affected by items of other colors. By contrasting the subset-specific slopes we found that the search became more selective toward items sharing the target color when the SOA between the neutral postcue and search array was increased, and an optimal level was reached after about 400 ms. Based on these results we suggested that it takes about this 400 ms for a visual WM template to maximally guide search when visual priming is controlled for.

A first question that these results raise is if visual search is guided as efficiently when based on a cued visual memory as when it is based on the visual cue itself. This is suggested by the similar time course as found in our study, compared to some studies that used a direct pictorial cue (Meyers and Rhoades, 1978; Schmidt and Zelinsky, 2011). However, it is impossible to conclude similar efficiency based on these studies, as their methods differed considerably in many aspects, including the stimuli, and the response requirements. Therefore, the aim of Experiment 1 was to directly compare the efficacy and time course of guidance initiated by a neutral postcue that pointed to a memorized target feature vs. a postcue that actually showed that feature.

The second aim was to re-investigate the role of visual vs. categorical precues in the formation of a memory template, but now under conditions that controlled for direct priming. In Experiment 2 we looked at whether postcued activation of a memory template leads to a distinct search guidance pattern when the precued template is formed after visual information vs. when it is formed after categorical (verbal) information. Such an effect would be predicted by previous cued search studies (Wolfe et al., 2004; Vickery et al., 2005; Schmidt and Zelinsky, 2011; Knapp and Abrams, 2012), but those studies did not control for visual priming effects that may stem from visual cues only. For these purposes, postcueing procedure was combined with a visual search task, in which accuracy performance and associated subset-specific slopes were measured.

## Experiment 1: a direct visual cue vs. a cued memory template

In Experiment 1 we investigated the time course of visual search guidance as based on either visual cues that include the target feature, or postcues that probe the memorized target template. Figure 1B shows the postcue manipulation that was used in the experiment. The effects of a neutral postcue were compared with those of an exact postcue that carried the relevant color. Both neutral and exact postcue were always valid (i.e., they always indicated the target color). One possibility is that seeing the color just before the search improves search performance and increases selectivity to that color, compared to probing the memory template of the same color. If the effects of the preview are due to rapidly emerging priming, the differences between the postcue types should be apparent already at the shortest cueing intervals. Alternatively, if search can be guided similarly by mere activation of a visual WM template, and if the potential effects of priming are negligible or redundant, there should be no difference between the neutral and exact postcue conditions.

In the current experiments we measured accuracy instead of RT as an index of search performance. The latter measure is more typical for visual search studies in general. However, there has been a large variability in the reported time it takes to implement guidance, when RT tasks were combined with SOA manipulation, with estimates varying between 200 and 800 ms (Meyers and Rhoades, 1978; Wolfe et al., 2004; Vickery et al., 2005; Knapp and Abrams, 2012). Part of this may be caused by the fact that RTs do not only reflect search itself, but are also affected by other processes such as response selection and execution. Therefore, search RT *slopes* (as a function of set size) have often been taken as a more direct index of guidance, as they reflect the average time spent on processing each item in the display. However, those cueing studies that measured RT slopes as a function of SOA have found either inconsistent, or no effects at all, of SOA or cue modality on this more direct measure of search guidance (Wolfe et al., 2004; Vickery et al., 2005; Knapp and Abrams, 2012).

In fact, there may be a more fundamental problem when RT tasks are used to estimate the time course of search guidance. As pointed out by Wolfe et al. (2004), because viewing time (and thus also response time) to the typical search display is in essence unlimited, observers can withhold the search until the template is fully set up and selectively implemented for the target feature—regardless of SOA. This would then result in efficient search (i.e., small slopes) irrespective of the cueing interval. Results from Wilschut et al. (2013) support this proposal. We found that search slopes in an unlimited duration RT task showed high selectivity for the target feature as early as 50 ms after the cue, and remained at a constant level irrespective of the SOA. In contrast, when the presentation of the search array was severely limited, and accuracy was measured instead, a gradual increase in search guidance over time was found. Accuracy-based search slopes suggested that selectivity in visual search was now enhanced up to 400 ms, after which it either stabilized or even declined again. This is well in line with another study that, instead of RTs, measured saccadic accuracy for search targets and found such accuracy to be enhanced up to about 400 ms after a visual cue, after which performance declined again (Schmidt and Zelinsky, 2011). Therefore, here too we measured accuracy as it appears to provide a more veridical estimate of visual search guidance as a function of time.

### Methods

#### Participants

Thirty students (7 male, 18–30 years, *M* = 21.7 years) participated in Experiment 1, and were compensated by money (8 €/h) or course credits. All reported normal or corrected-to-normal acuity and color vision, and gave their informed consent before starting the experiment. The data of six participants were replaced because five of these had a too low accuracy rate (<50% correct in average, 25% being chance level), and one preliminarily terminated the experiment because of experienced difficulties in the task. The exclusion criterion was the same as used in Wilschut et al. (2013), chosen to ensure that participants could stay sufficiently motivated. The results were however indifferent whether the five lower performing participants were included or excluded, as confirmed by additional statistical tests (not reported here). The experiments of this study were conducted in accordance with the Declaration of Helsinki, and the procedure was approved by the Scientific and Ethical Board of the Faculty of Psychology and Education of the VU University (VCWE).

#### Stimuli and apparatus

Participants were seated in dimly lit cubicles, approximately 70 cm from a 19-inch screen (1024 × 768 pixels, refreshing at 120 Hz). The stimuli were presented and data recorded by E-prime software (Psychology Software Tools Inc.). In all displays the background was gray (CIE: *x* = 0.308, *y* = 0.338, luminance: 29.95 cd/m^2^). A white (*x* = 0.279, *y* = 0.311, 110.9 cd/m^2^) circle with 0.1° radius marked the fixation. The precue consisted of a red (*x* = 0.627, *y* = 0.333, 6.9 cd/m^2^) and green (*x* = 0.304, *y* = 0.602, 9.8 cd/m^2^) circles, both with a 0.3° radius. The precue circles were centered 0.6° away from the fixation, 180° apart from each other, with the locations randomly varying across the trials. The postcue was a smaller circle (0.2°) centered at the location of one of the precue circles. In the *neutral postcue* condition, the circle was drawn black, whereas in the *exact postcue* condition it was drawn in the relevant target color. The search display consisted of 0.6° long lines that could be vertical, horizontal, or 45° tilted to the left or right. The lines were drawn in the same red and green as the precue circles. They were presented in the four quadrants of the array, with either 1 or 2 lines of both colors in each quadrant (in total 2 or 4 lines/quadrant, 8, 12, or 16 lines/array), depending on the set size. The item to item distance within quadrant was 1.9°, with an additional 1.2° between the quadrants. The whole area spanned thus an area of 8.6 × 8.6°. A mask was composed of 1500 randomly drawn red and green lines that covered the search array.

#### Procedure

The task is depicted in Figure 1B. Each trial started with the presentation of the precue for 1500 ms. After the precue and a 1000 ms blank period, the postcue was presented for 50 ms at the location of one of the precue circles. The postcue type (neutral or exact) was varied randomly within blocks. The search array followed the postcue at a randomly variable (50, 200, 400, and 1000 ms) stimulus onset asynchrony (SOA), and was covered by the mask for 300 ms. The search array duration was 150 ms for the first 26 participants. However, it turned out that six of these had severe difficulty performing the task (<50% correct and one quitting the task early), so we decided to add 10 more participants and increase the display duration to 167 ms. These 10 indeed performed at a sufficient level. Search array duration did not have a direct effect on the results or interact with any of the effects that are reported below. Fixation dot was present throughout the cue and search array presentation. When the participant had responded, there was a 1 s blank interval before the start of the next trial.

The task of the participant was to first memorize the locations of the two colors of the precue, and then select one of these as indicated by the postcue dot. Based on the cued color, participants then searched for one of the two predefined targets once the search array appeared. If the cued color was red, the target was always a red vertical line, and if the cued color was green, the target was a green horizontal line. Participants responded by pressing one of four keys on a standard keyboard based on whether they thought that the target was on the top left (“f”), top right (“j”), bottom left (“v”), or bottom right (“n”) quadrant of the search array. The instruction was to be as accurate as possible while the response speed was told to be unimportant. A sound feedback was given for each correct response, and mean accuracy was shown on-screen in the end of each block.

The postcue type (neutral vs. exact) and SOA (50, 200, 400, and 1000 ms) were varied within blocks. Also the set size of the lines appearing in the relevant, cued color, as well as the set size of the lines appearing in the irrelevant, uncued color were systematically varied (both 4 vs. 8 items) within blocks. This allowed us to calculate the search slopes independently for the relevant and irrelevant set, with higher search slopes indicating a more selective search among that color (cf. Egeth et al., 1984; Kaptein et al., 1995). Specifically, the slope was calculated as the increase in errors per each additional search item, separate for relevant and irrelevant color. The more observers are tuned toward the relevant color, the more search should be affected by items presented in that color, and the less by items in the irrelevant color. There were 20 trials per condition, resulting in 640 trials in total per each participant. The experiment included a practice block and 10 experimental blocks that together with self-determined breaks took about 70 min on average to complete.

### Results and discussion

**Figure 2:**
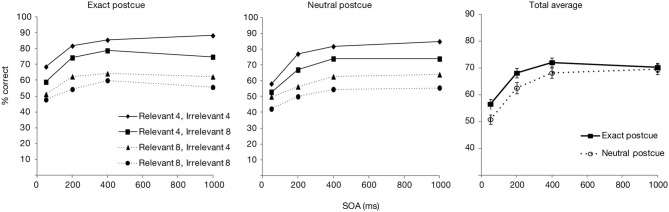
**Accuracy scores for Experiment 1, presented as a function of postcue type and SOA**. Left part of the figure shows accuracy scores separately for the relevant and irrelevant set sizes, right part shows the total averages over the set sizes. Error bars show the standard error of the mean here and in other figures.

Only the trials from the experimental blocks were analyzed. The trials in which responses were very fast (<200 ms) or slow (>4000 ms) were further excluded (0.9%). Figure 2 shows the overall accuracy score as a function of postcue type (neutral or exact) and SOA, as well as separated for the relevant and irrelevant set sizes. The data averaged over the set sizes was entered in an ANOVA with the postcue type and SOA as factors. Overall accuracy was significantly affected by both SOA [*F*_(3, 87)_ = 143.3, *p* < 0.001] and postcue type [*F*_(1, 29)_ = 34.7, *p* < 0.001]. Also the interaction effect was significant [*F*_(3, 87)_ = 3.0, *p* < 0.05]. *T*-test comparisons showed that with both postcue types, accuracies rapidly improved for the first two SOA increments (from 50 to 200 ms, and from 200 to 400 ms; all *p*s < 0.001), while after 400 ms an asymptote appeared to be reached (from 400 to 1000 ms: *p*s > 0.12). Performance for the exact postcue condition was significantly more accurate compared to the neutral postcue condition at all SOAs including 400 ms SOA (*p*s < 0.01 for all comparisons), after which the accuracy in the two conditions converged (*p* = 0.58 at 1000 ms).

**Figure 3:**
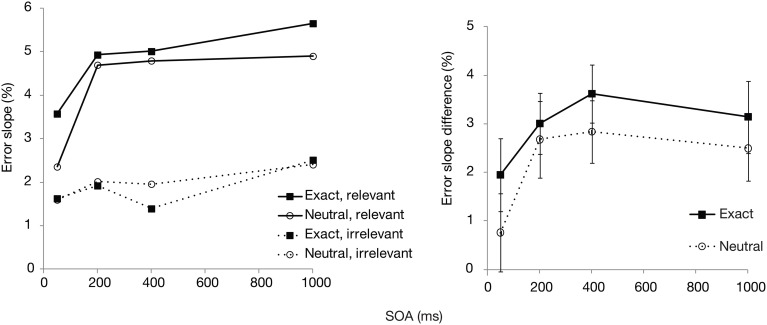
**Experiment 1: Mean error slopes for the relevant and irrelevant set, and their difference, plotted as a function of postcue type and SOA**.

We then analyzed the errors according to relevant vs. irrelevant set size, to see how SOA and postcue type may influence the *selectivity* in search. For this purpose, search slopes were calculated independently across the relevant (cued) color set size and irrelevant (uncued) set size. The difference between these two slopes (i.e., the subtraction of the latter from the former) was then used as an index of selectivity, with higher values showing that additional items in the cued color affected search more than additional items in the uncued color. These error slopes and their difference scores (relevant—irrelevant set) are plotted in Figure 3. Selectivity was significantly affected by SOA [*F*_(3, 87)_ = 2.9, *p* < 0.05]. Although there was a slight trend toward overall somewhat better selectivity in the exact cue condition, the effect of postcue type did not reach significance [*F*_(1, 29)_ = 2.2, *p* = 0.15]. There was also no interaction between the postcue type and SOA [*F*_(3, 87)_ = 0.2, *p* = 0.92], indicating a very similar time course for both postcue types. For both cues, selectivity increased up to 200–400 ms, after which it remained unchanged or, if anything, slightly declined.

To ensure that the accuracy scores, and also the derived selectivity measures were not distorted by scale restrictions, we arcsine transformed the raw accuracies and performed the same analyses again for these transformed values. The results with the transformed data were essentially identical. SOA and postcue type had significant effects on accuracy [*F*_(3, 87)_ = 135.0, *p* < 0.001; *F*_(1, 29)_ = 32.4, *p* < 0.001, respectively], and also the interaction effect was significant [*F*_(3, 87)_ = 3.1, *p* < 0.05]. Selectivity measures derived from the arcsine transformed data were significantly affected by SOA [*F*_(3, 87)_ = 3.9, *p* < 0.05], whereas the effect of postcue type [*F*_(1, 29)_ = 3.1, *p* = 0.09] and interaction [*F*_(3, 87)_ = 0.12, *p* = 0.95] were not significant.

The results of Experiment 1 show that seeing the target color just before the search improved target detection accuracy relative to a condition in which no such preview was present, and only a WM representation of the feature was available. The benefit was present right from the earliest SOA of 50 ms, remained significant up to 400 ms and dissipated thereafter. Such a rapid enhancement is consistent with the results of Wolfe et al. (2004) and Vickery et al. (2005) who showed that an exact cue improved search RTs already at the shortest SOAs. Importantly, this effect of the visual cue goes beyond the influence that the WM representation has on guidance.

Interestingly though, *selectivity* for the cued color was relatively unaffected by the color preview. Even though the exact postcue seemed to lead to slightly higher selectivity, this effect was not statistically significant. Furthermore, selectivity increased similarly as a function of time for both postcue conditions. The lack of finding significant effects with null-hypothesis testing may however be due to a Type II error. Therefore, we tested the main effect of postcue type and its interaction with SOA with regards to selectivity also by calculating Bayes factors. Bayesian statistics can provide evidence for or against a null-hypothesis. We transformed the sum-of-square values from the ANOVA with selectivity as the dependent variable, by using the routine provided by Masson (2011). Under the null-hypothesis the Bayes factor was 1.9 for the postcue effect, constituting little evidence for or against the null hypothesis of a lack of a main effect. Importantly, the Bayes factor for the interaction effect between the postcue type and SOA under the null-hypothesis was 152. This is very strong indication that the performance *time course* for the two postcue types did not differ. Therefore, although the overall accuracy showed that the exact postcue was more beneficial for detecting the search target, this preview benefit could not be found in the selectivity measure. Note that in the study of Wolfe et al. such a distinction could not be uncovered, as they looked at overall RTs and slopes across all search items.

An interesting question is then why the exact postcue was found to improve only the overall accuracy and not selectivity. We have suggested that some of the benefit by the visual cues could be based on automatic priming of the target. Inter-trial priming may speed RTs by facilitating different processing stages, namely perceptual, attentional (i.e., weighting of selection settings), or post-selection decision and response selection processes (Olivers and Meeter, 2006; Kristjánsson and Campana, 2010). As argued, given that the exact cue did not affect the relevant and irrelevant set slopes, it is unlikely that priming affected selectivity. However, as the responses were unspeeded, and accuracy was measured, it is also unlikely that the current benefits are caused at response selection stages. What remains then is that perceptual processing either prior to or after selection is speeded. For example, priming with the correct color may speed up the identification of a search item, or the decision to either accept a selected item as relevant or reject it as irrelevant.

To test whether such an explanation could in principle account for the current data, we explored how perceptual processing efficacy modifies overall accuracy and selectivity by building a relatively simple model. The model simulates target detection probability as a function of stimulus processing time—specifically, the time spent per search item. In the model, detection probability *p_d_* is determined individually for each set size combination (i.e., of relevant and irrelevant items) by:

(1) $$p_{d}\text{ = }S_{\text{rel}}/\left(N_{\text{rel}}\times 0.5\right).$$

where *S*_rel_ refers to the number of relevant items that can be sampled during the trial, given the processing speed. *N*_rel_ refers to the number of all relevant items in the display, and on average half of these need to be sampled before the target is found. With adjustment for the 25% probability guessing rate, the probability of a correct response, *p*_corr_ becomes:

(2) $$p_{\text{corr}}\;=\;p_{d}\;+\;\left(\text{1 }-p_{d}\right)\;\times \text{ 0}.\text{25}.$$

The number of relevant items that can be sampled (*S*_rel_) during a display presentation is determined by (a) the total number of items that can be sampled during the brief display presentation, *S*_tot_, and (b) the effective ratio of the number of relevant items over the number of irrelevant items. The latter is in principle determined by the sizes of the relevant and irrelevant sets, *N*_rel_ and *N*_irr_.

(3) $$S_{\text{rel}}\;=\;S_{\text{tot}}\;\times \;N_{\text{rel}}\;/(N_{\text{rel}}\;+\;N_{\text{irr}}).$$

However, to simulate the changing selectivity with time, *N*_irr_ is set to decrease with time, such that at each SOA, the number of effective irrelevant items is halved, except for the longest SOA (1000 ms), where we assumed no further change.

The important variable is then *S*_tot_, the number of items that can be sampled. For a fixed display duration *d*, this will be determined by the perceptual processing time of an item, *t*, such that

(4) $$S_{\text{tot}}=d\;/\;t.$$

**Figure 4:**
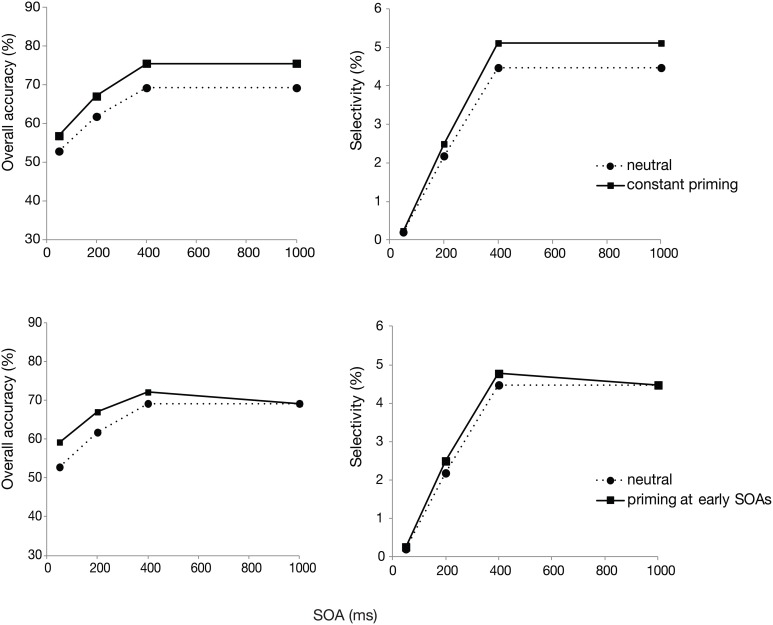
**Model simulations for overall accuracy and selectivity**. The top row shows a constant priming at all SOAs. The bottom row shows priming that decreases with SOA.

In the model, *d* is fixed at 160 ms. We then assumed that stimulus processing time *t* would be reduced by priming. Thus, all we varied was *t*. Figure 4 shows how simulated target detection probability, as well as selectivity changes according to two different priming scenarios. The overall probability of a correct response and selectivity are derived from the simulated detection probability values, thus calculated in the same way as for the real data. In both scenarios, stimulus processing time (*t*) was set to 80 ms for the unprimed (neutral postcue) condition, and thus serves as a reference in each plot. The top row then shows a model in which priming is *constant*, reducing stimulus processing time by 10 ms at every SOA. The bottom row shows a model in which priming is rapid and strong in the beginning, but dissipates with SOA. Here, stimulus processing time *t* is set to 65, 70, 75, and 80 ms for SOAs of 50, 200, 400, and 1000 ms, respectively.

Comparing the plots of the model simulations to the observed data (see Figures 2, 3) suggests a good qualitative fit for the case where the stimulus processing time is only temporarily speeded by priming (bottom row of Figure 4). For this simulation, with priming occurring only at early SOAs, the overall probability of correct response is enhanced for the first 400 ms, but selectivity seems to be little affected, similar to what was observed in the current experiment. In contrast, reducing the stimulus processing time in a constant manner (top row of the figure) results in the strongest modulations at the latest SOAs, contrary to what we found. The model thus, provides support for the idea that priming enhances perceptual processing or perceptual decision making, but that this priming effect lasts only for the first few 100 ms after the cue.

## Experiment 2: categorical vs. visual precues

Our next question was whether visual search is more efficient when the memory template is based on a visual cue than when it is based on a categorical cue. Earlier studies have shown that *presenting* a categorical cue is less effective than *presenting* a visual cue, but does the same go for a memorized cue? We tested this by presenting two possible target colors in either visual or verbal form (see Figure 1C). Then a neutral (black) postcue indicated on every trial which of the two colors was the target color. Note that through this procedure, direct visual priming is controlled for, since (a) both the relevant and the irrelevant color are presented on a trial, and (b) the search display is timed not to these colors, but to the postcue, which does not carry any task-relevant color. We again manipulated the SOA between the postcue and the search array to investigate how search performance would develop over time as a function of either visually or categorically initiated templates in WM.

### Methods

The task and methods were as in Experiment 1, except for the changes described below.

#### Participants

Twenty-nine students (10 male, 18–29 years, *M* = 22.5 years) participated in Experiment 2, fulfilling the same criteria and receiving compensation as in Experiment 1. The data of two participants were replaced due to lower than 50% mean accuracy.

#### Stimuli and procedure

The stimuli were as in the neutral postcue condition of Experiment 1, except that now the type of precue was manipulated (see Figure 1C). Visual precues consisted of the red and green circles, as in Experiment 1. Categorical precues consisted of words “red” and “green,” printed in capital, black, size 12 Helvetica font, presented in the participant's mother tongue (as they typed in themselves at the beginning of the experiment). The disks or words were centered 1° away from central fixation, preventing the words from overlapping. The postcue was always a black dot. The search array duration was 167 ms.

Precue type was thus either categorical (words) or visual (colored circles), which was manipulated between blocks in an alternating fashion (A-B-A-B). Half of the participants started with the categorical condition and the other half with the visual condition. SOA (4 levels) as well as relevant and irrelevant set size (both 4 vs. 8) were varied within blocks. There were 20 trials per cell in the design (640 trials in total) for each participant. The task was again to search for the target bar defined by the cued color, and indicate in which of the four quadrants it was.

### Results and discussion

**Figure 5:**
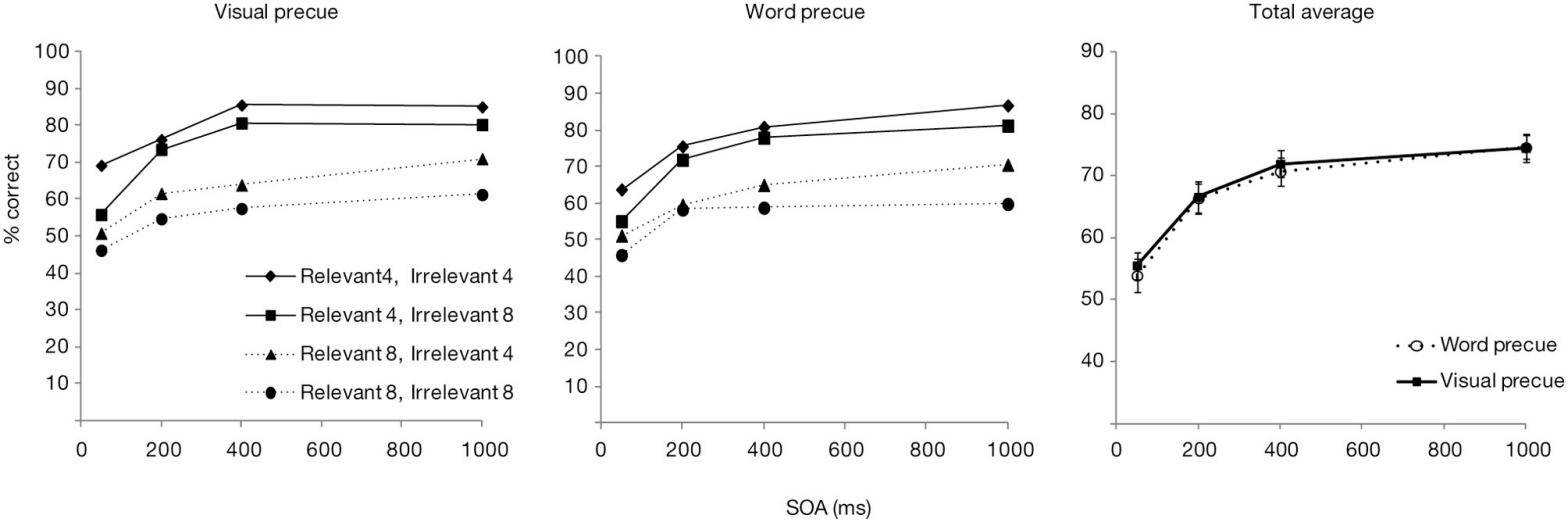
**Accuracy scores for Experiment 2, presented as a function of precue type and SOA**. Left part of the figure shows accuracy scores separately for the relevant and irrelevant set sizes, right part shows the total averages over the set sizes.

Of all trials, 0.8% was excluded because of extremely fast or slow (<200 or >4000 ms) responses. The pattern of overall accuracy as a function of SOA and precue type, as well as according to relevant and irrelevant set sizes are shown in Figure 5. The figure suggests that accuracy improved when the SOA was increased, and that this was identical for the two precue conditions. A repeated measures ANOVA confirmed these observations, by showing a significant effect of SOA [*F*_(3, 84)_ = 128.7, *p* < 0.001] but no effect of precue type [*F*_(1, 28)_ = 0.83, *p* = 0.37], nor an interaction of the two factors [*F*_(3, 84)_ = 0.33, *p* = 0.81]. *Post-hoc t*-tests indicated that accuracy improved significantly at every SOA increment in both precue conditions, except for the increment from 400 to 1000 ms when the precue was visual (*p* = 0.1; for all other comparisons: *p*s < 0.01). When performance between the two precue conditions was compared at each SOA, there were no differences (all *p*s > 0.23).

**Figure 6:**
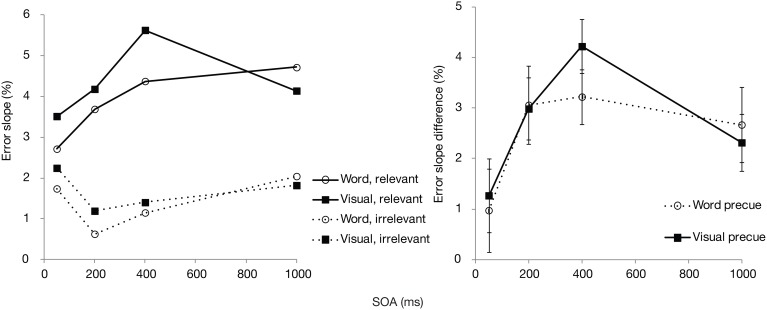
**Experiment 2: Mean error slopes for the relevant and irrelevant set, and their differences, plotted as a function of precue type and SOA**.

Selectivity scores (subset-specific slopes for relevant and irrelevant colors, and their differences) are shown in Figure 6 for each precue type and SOA separately. Similar to the overall accuracy results, the measure of selectivity was significantly affected by the SOA [*F*_(3, 84)_ = 5.1, *p* < 0.01]. Even though the figure suggests a somewhat sharper enhancement and decline in selectivity for the visual precue, there was no statistically significant effect of the precue type [*F*_(1, 28)_ = 0.25, *p* = 0.62] or an interaction between the precue type and SOA [*F*_(3, 84)_ = 0.45, *p* = 0.72].

Again, to ensure that the result pattern was not an artifact arising from a measurement scale compression, we arcsine transformed the data and performed the analyses with the transformed values. For these data, overall accuracy scores were significantly influenced by SOA [*F*_(3, 84)_ = 138.3, *p* < 0.001], with no effect of precue type [*F*_(1, 28)_ = 0.64, *p* = 0.43] nor an interaction effect [*F*_(3, 84)_ = 0.23, *p* = 0.88]. The selectivity scores derived from the transformed data were significantly influenced by SOA [*F*_(3, 84)_ = 6.6, *p* < 0.001], whereas the effect of precue type [*F*_(1, 28)_ = 0.1, *p* = 0.76] and the interaction effect [*F*_(3, 84)_ = 0.29, *p* = 0.83] were not significant. The results thus replicated those based on raw accuracies.

Furthermore, to search more evidence for the lack of precue effect on selectivity, and the lack of interaction between the precue type and SOA, we transformed the results from the ANOVA to Bayes factors. Under the null-hypothesis, these were 4.7 and 124, positive and very strong evidence for no precue type effect nor interaction, respectively.

Thus, both precue modalities (visual and categorical) in Experiment 2 led to very similar estimates of search efficacy both in terms of overall accuracy and in terms of selectivity. Accuracy was significantly improved as the time between the postcue and search array increased, with still some significant improvement until the longest SOA of 1000 ms. Selectivity for the cued color increased up to 400 ms, after which it did not rise further, or even dropped (in the visual precue condition). This performance pattern is consistent with what we have found earlier, when only a visual precue type was used (Wilschut et al., 2013). Interestingly, the finding that selectivity for the relevant feature was enhanced in a similar fashion with both visual and categorical precues is in contrast with the previous studies that have found strong guidance for visual cues only (Wolfe et al., 2004; Vickery et al., 2005; Schmidt and Zelinsky, 2011; Knapp and Abrams, 2012).

There are several possible factors that may contribute to these deviating results. First, selectivity of visual search was measured here directly as the extent to which search was performed among items of the cued feature, whereas the measures used in other studies have varied from basic RTs to saccadic accuracy to the target. As sketched in the introduction of Experiment 1, especially RTs may not be ideal for successfully capturing the changes in visual search guidance over the cueing intervals. Second, it is possible that the rapid and strong guidance enhancement as has been found for visual cue types only in the previous studies was boosted by direct priming type of activation from the cue. In the current experiment we controlled for such automatic priming effects by measuring changes as a function of a neutral postcue.

Another possibility is that the template was in fact the same in both precue type conditions. Note that although the presentation of the precue differed between visual and categorical, this does not mean that the eventual memory representation differed too. It is possible that observers, when seeing the visual color disks, transformed these into a categorical code. Note however, that this would mean sacrificing performance, as previous work has suggested better guidance on the basis of visual representations. Alternatively, it is possible that the color words were turned into a visual WM representation that is then just as effective as a representation based on visual input. The interesting question then rises as to why this interchangeability occurs for memories, while it does not occur for categorical and visual cues that are actually presented as such. Note that visual and categorical cues may not have been encoded in the corresponding modalities in the previous studies either, but these found robust differences between the cue types. The point that we want to make here is that WM templates based on visual input are no more effective than WM templates based on categorical input. This suggests that previous advantages of visually presented cues were mainly due to direct visual priming, rather than loading the visual representation into WM.

## General discussion

This study investigated the time course of visual search guidance as based on memory templates formed from various cue types. Earlier studies on this topic have resulted in diverse findings that may be in part attributed to the methods used. Most importantly, the cues that have been assumed to evoke a visual WM representation may instead, or in addition, have activated more automatic types of visual memory. Here we aimed to resolve whether visual search guidance differs between an exact visual cue that directly shows the target feature, and a cue that points to a memory template containing that feature. We also investigated how search is guided by templates that are formed based on either categorical or visual information. Experiment 1 showed that exact visual cues improved overall search accuracy relative to cues that activated a target template that was no longer visible but maintained in WM. However, no such benefit was found for selective search among relevant (cued) vs. irrelevant (uncued) items.

Whereas previous work compared the effects of presented categorical and visual cues, Experiment 2 compared the effects of *memorized* categorical and visual cues, while cue-target priming was controlled for. In contrast to the previous work, we found no differences in either search accuracy or selectivity.

The general pattern found in the current experiments was that cueing a visual WM template enhanced search guidance progressively, so that the highest level of selectivity was reached at about 400 ms after the cue. This is in line with what has been shown by other cued visual search studies that used accuracy-based measures (Schmidt and Zelinsky, 2011; Wilschut et al., 2013). Similar timing was also found in an initial study that measured pure RTs (Meyers and Rhoades, 1978), whereas more recent RT studies have suggested either faster (Wolfe et al., 2004; Vickery et al., 2005) or slower (Knapp and Abrams, 2012) times for search guidance to develop. As was shown by our earlier study (Wilschut et al., 2013), RT tasks may however not best capture the gradual build-up of search guidance over time. The current results are in line with findings from the two other studies that used accuracy-based measures (Schmidt and Zelinsky, 2011; Wilschut et al., 2013), and show that it takes about 400 ms for a visual WM representation to guide visual search efficiently.

### Visual search guidance based on exact visual cues and cued memory templates

Importantly, the present results show that visual search can be guided efficiently based on representations that are in WM, as has been proposed by various theories of visual attention (Duncan and Humphreys, 1989; Bundesen, 1990; Wolfe, 1994; Desimone and Duncan, 1995). Note that this has been doubtful for studies that presented the search displays time-locked to a presentation of the target image, as in these performance could have been influenced by sensory processing (visual encoding) of the cue (Wolfe et al., 2004; Vickery et al., 2005; Schmidt and Zelinsky, 2011; Knapp and Abrams, 2012). Experiment 1 demonstrated that selectivity for the target color improved at similar speed and extent when based on cued memory as when the color was shown just before the search. This rules out the possibility that guidance toward the target color would be explained merely by priming, instead of relying on WM-based top-down control. Even though the more automatic types of representation may guide search in some situations (Wolfe et al., 2003, 2004), it is important to show that search for objects or features that are activated within WM can also be rather fast and effective.

Still, we did find benefits for the exact cues that were evident already at the shortest SOAs and lasted for 400 ms. However, this was a benefit on overall accuracy, rather than guidance. The fact that this was an overall and not a selective effect may also explain why previous cueing studies found rapid and strong improvement of overall RTs by exact visual cues, but the effects on RT slopes were small and inconsistent (Wolfe et al., 2004; Knapp and Abrams, 2012). Exact cues seem to prime target search, but specifically through facilitating overall performance measures. The current findings then serve as another reminder that different measures (RT, accuracy, search slopes, and even search slopes for subsets of items) are sensitive to different aspects of the task, and may thus lead to different conclusions based on the same overall experimental design. We believe that by looking at subset-specific effects we have a more precise handle on attentional guidance than was previously the case. Furthermore, Experiment 1 suggests that such measure can dissociate guidance from other effects, such as priming.

Which aspects of processing are then influenced by priming, as is evident in the overall accuracy measure of our task? Given that response accuracy was affected while selectivity was not, we assumed that the priming effect would have a perceptual locus. In support of this, model simulations showed that an early priming effect on overall accuracies plus no effect on selectivity could be reproduced by varying the processing time of the search items. Specifically, model behavior resembled closely the pattern of results of the experiment when priming was assumed to reduce stimulus processing time at the shortest SOAs. The results are thus in line with the idea that priming improves visual search task performance by enhancing perceptual processing of the cued items. Such an enhancement may comprise of boosting the target signal prior to selection (Lee et al., 2009; Meeter and van der Stigchel, 2013), and speeding perceptual decision about the target identity once it is already selected (Huang et al., 2004). While the current results may not differentiate between these two possibilities, they nevertheless show how exact cues prime the perceptual processing of the search target and thereby influence measures of visual search task performance.

### Visual search guidance by categorical and visual cues

Interestingly, we failed to find any support for the weak and slow type of visual search guidance by templates based on categorical cues, as shown by other cued search studies (Meyers and Rhoades, 1978; Wolfe et al., 2004; Vickery et al., 2005; Schmidt and Zelinsky, 2011; Knapp and Abrams, 2012). In our task categorical and visual precues led to identical search performance in terms of both time course and amplitude. One reason for this could be that we presented the cues well in advance so that WM representations could be encoded, and measured performance as a function of a postcue that indicated which color had to be activated in memory. This avoided the beneficial effect of target priming by the visual cue. It also avoided the costs associated with reading and encoding the categorical word cue, which is likely to have delayed search in earlier studies. However, differential encoding of visual and categorical cues may not be the only source of the visual cue advantage, as this has previously been observed to last for more than 6 s after the cue (Knapp and Abrams, 2012). Such an interval should be long enough for successful encoding of even the categorical WM representation. The visual cue benefit could then be still due to visual priming, which has been suggested to last at least tens of seconds (Maljkovic and Nakayama, 2000; Thomson and Milliken, 2012). Note that in our Experiment 1, the benefits of the visually presented postcue were over after 400 to 1000 ms, but this was when comparing it against a visually initiated WM. Moreover, ceiling effects may have prevented further differential effects at the long SOAs, as overall performance reached asymptote.

A second possibility could be that the differences that were found between the cue modalities in previous studies may not necessarily reflect search guidance but some other task-related process. Again, most evidence for these differences comes from basic RT measurements (Meyers and Rhoades, 1978; Wolfe et al., 2004; Vickery et al., 2005; Knapp and Abrams, 2012), which could be influenced by pre- or post-selective processes. Still, clear visual cue benefits were also found by Schmidt and Zelinsky (2011) who used saccadic accuracy as a measure of guidance. This should not suffer from the problems that are inherent in RT measurements, and may be more analog to the accuracy measure in the present study. Therefore, the measurement type does not seem to fully explain the differences either.

Third, it may be that we found equally efficient search by categorical and visual cues because simple color features were used, instead of visually more complex targets (e.g., real-life objects) as used by previous investigators. Such simple features may be easier to encode into both visual and categorical WM templates. Visual WM can allow for encoding several features and details concurrently, within the same representation (Luck and Vogel, 1997), whereas encoding in categorical WM is thought to be serial and thus more restricted at a given moment (Baddeley, 1992). Therefore, especially the studies that have used complex and/or realistic images as targets may have been prone to find superior search guidance by visual cues compared to categorical, as the former can lead to richer representation (Wolfe et al., 2004; Vickery et al., 2005; Schmidt and Zelinsky, 2011). This is in line with the idea that visual search is limited in the specificity of target representation, with more precise target representations leading to more effective search (Schmidt and Zelinsky, 2009). The current study (see also Anderson et al., 2010 for supporting evidence) suggests that if the informational content is equal between categorical and visual cues, these can lead to guidance of visual search in a comparable time course and efficiency.

As we have pointed out in the discussion of Experiment 2, one could also argue that the visual and categorical cues were not encoded into visual and categorical WM, respectively. Instead, visual cues may have been encoded categorically, or categorical cues may have been encoded visually. A more strict way to enforce for visual representation would be to use target features that are difficult to verbalize (Olivers et al., 2006) or to prevent subvocal rehearsal during encoding (Soto and Humphreys, 2008). In addition, to ensure that categorical cues were not encoded visually, an analog visual control may be needed (e.g., prevention of “visual rehearsal” by presenting irrelevant visual stimuli). Future studies are needed to control for the mode of encoding or maintenance. In the meantime, the current results stand in contrast to previous studies in showing that visual search is guided at similar time course and intensity by templates in WM that have been initiated by either categorical or visual cues.

Since our procedure allows sufficient time for encoding the precue (regardless of modality), the current time course most likely reflects the time that is needed in order to turn the already existing WM representation from a “standard” memory into a search-guiding template. As proposed by Olivers et al. (2011), a set of WM representations, when not yet task relevant, may be initially maintained in a passive mode which does not influence visual perception. Only when one of them is pressed into service of the search task, for example by integrating information about task instructions, it will turn into a fully operative and selective attentional template. In line with this idea, a recent study (Wolfe et al., 2010) showed that search became maximally selective about 200 ms after the search display onset even in blocks in which the target was always the same. Therefore, even though the same target representation could be constantly active, and should already be on the observer's mind, there was still an additional few 100 ms needed to put it into a mode that is useful for search. Thus, when the type and modality of the target representation have a relatively minor role, the time course of visual search guidance seems to be mainly determined by the time needed to create a template out of a memory.

### Conclusions

This study shows that visual search can be guided at a comparable time course and efficacy when based on exact visual cues that *showed* the target feature, and when based on cued memory templates that *maintained* the feature. However, overall response accuracy was found to be enhanced by exact cues, suggesting a priming benefit at perceptual stage. Furthermore, the study suggests that when the effects of direct visual cues are controlled for, search guidance is identical when performed by visual and category-based target templates, in contrast to the results of previous investigations using visual cues rather than memorized cues. The results further underline the importance of carefully selecting the methods and measures to investigate visual search guidance as a function of WM templates and time.

### Conflict of interest statement

The authors declare that the research was conducted in the absence of any commercial or financial relationships that could be construed as a potential conflict of interest.
